# Chatbots for Smoking Cessation: Scoping Review

**DOI:** 10.2196/35556

**Published:** 2022-09-26

**Authors:** Robyn Whittaker, Rosie Dobson, Katie Garner

**Affiliations:** 1 National Institute for Health Innovation University of Auckland Auckland New Zealand; 2 Waitemata District Health Board Auckland New Zealand

**Keywords:** chatbot, conversational agent, COVID-19, smoking cessation

## Abstract

**Background:**

Despite significant progress in reducing tobacco use over the past 2 decades, tobacco still kills over 8 million people every year. Digital interventions, such as text messaging, have been found to help people quit smoking. Chatbots, or conversational agents, are new digital tools that mimic instantaneous human conversation and therefore could extend the effectiveness of text messaging.

**Objective:**

This scoping review aims to assess the extent of research in the chatbot literature for smoking cessation and provide recommendations for future research in this area.

**Methods:**

Relevant studies were identified through searches conducted in Embase, MEDLINE, APA PsycINFO, Google Scholar, and Scopus, as well as additional searches on JMIR, Cochrane Library, Lancet Digital Health, and Digital Medicine. Studies were considered if they were conducted with tobacco smokers, were conducted between 2000 and 2021, were available in English, and included a chatbot intervention.

**Results:**

Of 323 studies identified, 10 studies were included in the review (3 framework articles, 1 study protocol, 2 pilot studies, 2 trials, and 2 randomized controlled trials). Most studies noted some benefits related to smoking cessation and participant engagement; however, outcome measures varied considerably. The quality of the studies overall was low, with methodological issues and low follow-up rates.

**Conclusions:**

More research is needed to make a firm conclusion about the efficacy of chatbots for smoking cessation. Researchers need to provide more in-depth descriptions of chatbot functionality, mode of delivery, and theoretical underpinnings. Consistency in language and terminology would also assist in reviews of what approaches work across the field.

## Introduction

### Background

Tobacco use is the single most preventable cause of premature deaths worldwide. Tobacco use kills over 8 million people every year and is a risk factor for cancer, respiratory disease, cerebral vascular disease, heart disease, and a number of other debilitating chronic diseases [1]. Quitting smoking can lower the risk of some tobacco-related illnesses, and in some cases, ex-tobacco users can gain years in life expectancy compared to those who continue to use tobacco. Despite significant progress over the past 2 decades, with global tobacco use falling from 1.397 billion in 2000 to 1.337 billion in 2018 or approximately 60 million people [2], many countries are still not adequately implementing policies that can save lives from tobacco use, and the global target set by governments to reduce the prevalence of tobacco use by 30% by 2025 remains off track. One of those policies is World Health Organization (WHO) Framework Convention on Tobacco Control (FCTC) Article 14, which offers help to quit tobacco.

COVID-19 is an infectious disease that primarily affects the lungs, and tobacco smoking is a known risk factor for many respiratory infections and increases the severity of respiratory diseases. Early in the pandemic, the WHO convened a group of experts and found that severe COVID-19 is more likely to develop in smokers than nonsmokers. This news triggered millions of tobacco users to want to quit [3]. Despite approximately 60% of tobacco smokers voicing the desire to quit, only 30% have access to resources that will enable them to quit successfully [3].

Harnessing the power of digital solutions may be one approach to bridging this gap in the availability and delivery of cessation services globally. The subscription statistics of global telecommunications companies show that there are currently approximately 3 billion smartphone users, a number that is expected to grow by millions in the next few years [4]. This rapid growth in smartphone ownership and internet access has paved the way for new opportunities to reach tobacco smokers and provide smoking cessation support. Digital interventions have significant advantages for health care, such as flexibility, anonymity, cost-effectiveness, scalability, and increased access [5,6].

Previous research has found that SMS text messaging interventions are effective for helping tobacco users to quit successfully. Systematic reviews of high-quality (low-bias) randomized controlled trials (RCTs) of smartphone smoking cessation programs have found that SMS text messaging interventions generally double the success rate of abstinence [7,8]. A 2019 Cochrane review found beneficial results for 6-month cessation outcomes with text messaging, while there was insufficient evidence at that point for smartphone app interventions [8].

Most SMS programs use behavior change techniques (BCTs), which have been found to contribute to intervention efficacy [9]. Techniques from the COM-B model, which describes that a person’s capability, motivation, and opportunity all contribute to their behavior [10], such as “facilitate relapse prevention and coping,” “provide information on the consequences of smoking and smoking cessation,” “promote the use of relaxation techniques,” and “advise on/facilitate the use of social support,” can be easily integrated into messaging interventions [9].

These BCTs that have been proven effective could also be used in chatbots or conversational agents, which are digital tools that allow users to talk with the program through voice or text [11]. These tools are designed to mimic human communication and provide friendly and engaging answers to directly respond to the user’s questions or concerns [12]. In recent times, chatbots have been used in health care for supporting clinicians in diagnosis and helping individuals manage chronic illnesses [13]. Because chatbots allow instantaneous feedback and are widely available through social media and messaging apps, such as Facebook Messenger, it is hypothesized that they may be particularly suited to support smoking cessation, building on previous text messaging research.

During the COVID-19 pandemic, the WHO developed several smoking cessation chatbots, using content from existing smoking cessation programs. These were launched in response to the WHO initiative to get 100 million people to quit smoking, and they include Florence (a virtual human) and chatbots delivered on Viber, WhatsApp, Facebook Messenger, and WeChat, although formal evaluations of these are not yet available.

### Current Review

Despite the current potential of chatbots to help tobacco users quit, there are no reviews to assess their efficacy among adult smokers. The purpose of this scoping review is to assess the efficacy and summarize the research on chatbots for smoking cessation. The review includes a search of published and grey literature to examine the full extent of evidence.

## Methods

### Identification of Studies

This review was guided by the PRISMA-ScR (Preferred Reporting Items for Systematic Reviews and Meta-Analyses Extension for Scoping Reviews) checklist [14], and the protocol was not registered. Embase, MEDLINE, APA PsycINFO, Google Scholar, and Scopus were searched on July 2, 2021. We also ran an additional search on JMIR, Cochrane Library, Lancet Digital Health, and Digital Medicine to identify any more relevant articles. An iterative process was used to develop the search strategy. Words associated with smoking cessation were combined with words for chatbots or “conversational agents” (see Textbox 1 for all search terms for each database). These combinations were used to create search strings, which were then put into the databases. Filters were used to limit the search to articles published between 2000 and 2021. The reference lists of included articles were also searched for any new studies that met the inclusion criteria.

**Search terms.**

**Embase**

1. smoking cessation.mp. or exp Smoking Cessation/

2. (((quit$ or stop$ or ceas$ or giv$ or prevent$) adj3 smok$) or cigarette$).ti,ab.

3. exp passive smoking/

4. exp smoking habit/

5. smokeless tobacco/

6. smoking reduction/

7. (smok* or tobacco).mp.

8. 1 or 2 or 3 or 4 or 5 or 6 or 7

9. (Conversational agent$ or embodied conversational agent or chatbot$ or avatar or dialog$ system or virtual assistan$ or virtual nurs$ or virtual patient or virtual coach$ or intelligent assistan$ or relation$ agent or assistance technol$ or voice-based interfac$ or virtual coach or speech recognition software or voice recognition software).mp. [mp=title, abstract, heading word, drug trade name, original title, device manufacturer, drug manufacturer, device trade name, keyword, floating subheading word, candidate term word]

10. 8 and 9

**MEDLINE**

1. smoking cessation.mp. or exp Smoking Cessation/

2. (((quit$ or stop$ or ceas$ or giv$ or prevent$) adj3 smok$) or cigarette$).ti,ab.

3. exp passive smoking/

4. exp smoking habit/

5. smokeless tobacco/

6. smoking reduction/

7. (smok* or tobacco).mp.

8. 1 or 2 or 3 or 4 or 5 or 6 or 7

9. (Conversational agent$ or embodied conversational agent or chatbot$ or avatar or dialog$ system or virtual assistan$ or virtual nurs$ or virtual patient or virtual coach$ or intelligent assistan$ or relation$ agent or assistance technol$ or voice-based interfac$ or virtual coach or speech recognition software or voice recognition software).mp. [mp=title, abstract, original title, name of substance word, subject heading word, floating sub-heading word, keyword heading word, organism supplementary concept word, protocol supplementary concept word, rare disease supplementary concept word, unique identifier, synonyms]

10. 8 and 9

**APA psychINFO**

1. smoking cessation.mp. or exp Smoking Cessation/

2. (((quit$ or stop$ or ceas$ or giv$ or prevent$) adj3 smok$) or cigarette$).ti,ab.

3. exp passive smoking/

4. smokeless tobacco/

5. smoking reduction/

6. (smok* or tobacco).mp.

7. 1 or 2 or 3 or 4 or 5 or 6

8. (Conversational agent$ or embodied conversational agent or chatbot$ or avatar or dialog$ system or virtual assistan$ or virtual nurs$ or virtual patient or virtual coach$ or intelligent assistan$ or relation$ agent or assistance technol$ or voice-based interfac$ or virtual coach or speech recognition software or voice recognition software).mp. [mp=title, abstract, heading word, table of contents, key concepts, original title, tests & measures, mesh]

9. 7 and 8

### Eligibility

Articles included in this scoping review met the following requirements: (1) conducted in tobacco smokers, (2) conducted between 2000 and 2021, (3) available in English, and (4) included a chatbot intervention. As this was a scoping review, we did not put any restrictions on study design; however, thesis manuscripts were excluded.

### Study Selection

Once duplicates were removed using EndNote, the titles were screened according to the inclusion criteria by 2 authors (KG and RD). The software used for this was Rayyan [15], a web program for systematic and scoping reviews. During this screening process, titles were screened to assess their relevance to the research question and criteria. If the relevance could not be inferred from the titles, the abstracts of the selected studies were reviewed according to the inclusion criteria. The next step was to assess the full text of articles that met the inclusion criteria and assess their relevance. Finally, the 2 researchers (KG and RD) met to discuss any disagreements and resolve any discrepancies. If necessary, a third researcher was consulted.

### Data Extraction and Synthesis

Two authors (KG and RD) extracted the data from the included studies on July 14, 2021. A predesigned spreadsheet was used to extract the data, including the title, author(s), total sample size, country, age mean and SD, intervention duration, theory referenced, measures, assessment timepoints, comparison condition, and main results, as well as whether a power calculation was conducted.

## Results

### Study Selection

**Figure 1:**
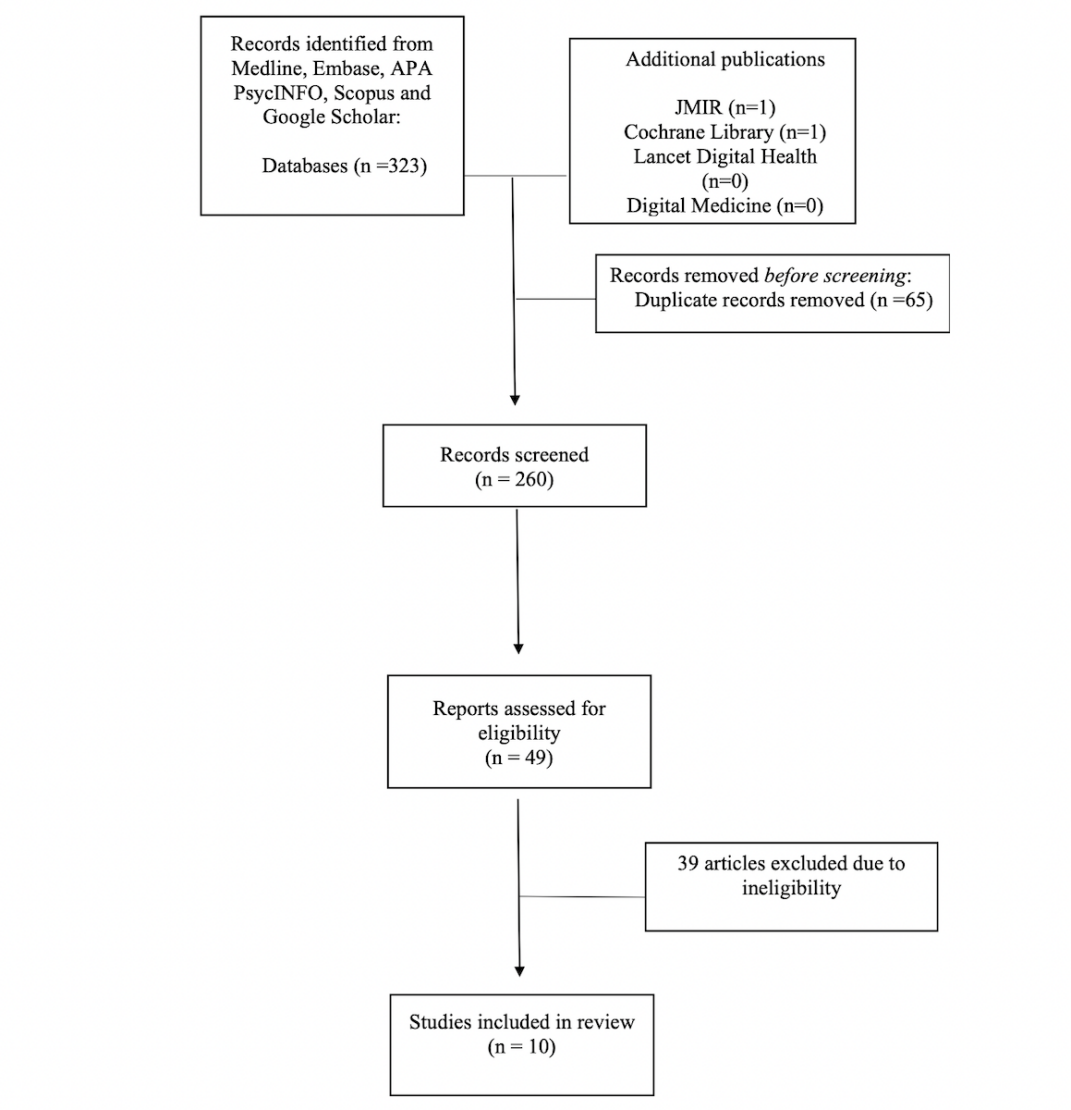
Flowchart of the literature search and article selection. JMIR: *Journal of Medical Internet Research*.

The database search identified a total of 323 studies, and an additional 2 studies were found from Cochrane Library and JMIR (Figure 1). After duplicates were removed, 259 articles were screened. After the final review, 10 studies were included. Studies were excluded for several reasons: (1) the conversational agent was actually a human communicating via social media, (2) the paper was a thesis, and (3) the intervention did not target smoking cessation specifically.

### Study Characteristics

There was a mix of 3 framework articles [16-18], 1 study protocol [19], 2 pilot studies [20,21], 2 trials [22,23], and 2 RCTs [24,25]. The studies included in this review are presented in Table 1, and further details are provided in Multimedia Appendix 1.

Three papers were predominantly descriptive and did not present any interventions [16-18]. These described an array of topics in the smoking cessation chatbot literature, including single-agent and multiagent chatbots based on cognitive behavioral therapy [16]; a description of designing an embodied conversational agent for smoking cessation [17]; and research, theory, and considerations for smoking cessation chatbots in adolescents with a low socioeconomic status [18].

**Table 1:** Studies included in the review.

Study	Type of paper	Chatbot intervention	Theory
Abdullah et al [22]	Study trial	Simple embodied conversational program that encourages smokers to think about setting a quit date. When participants log in, the agent initiates a discussion based on recorded information in the previous session.	N/A^a^
Almusharraf et al [23]	Study trial	Chatbot based on motivational interviewing. The chatbot delivers questions about the pros and cons of smoking.	Motivational interviewing
Avila-Tomas et al [19]	Study protocol	Chatbot that guides users through the stages of the dishabituation process. Includes cognitive-behavioral, relapse-prevention, and problem-solving techniques.	Gamification, cognitive behavioral theory, problem-solving
Calvaresi et al [16]	Framework paper	Describes a single-agent and multiagent chatbot based on CBT^b^ and MAS^c^ (underlying technology that models human-like behaviors).	CBT
Grolleman et al [17]	Framework paper	Describes designing an embodied conversational agent for smoking cessation.	Motivational interviewing and nonverbal listening techniques
Karekla et al [20]	Pilot randomized clinical trial	Digital avatar–led ACT^d^ smoking cessation program. The program ran for 6 sessions, 25 minutes each. The avatar provides questions, and users answer. Involved ACT-based activities and homework exercises.	ACT
Masaki et al [21]	Single-arm pilot study	Smoking cessation app called CureApp that includes an artificial intelligence nurse to which users can send a message when they have a craving or are going through withdrawal. The nurse will respond with personalized advice and how to deal with the symptom.	N/A
Perski et al [24]	Randomized controlled trial	Smoking cessation chatbot that guides users through the UK “Stop Smoking Services” standard smoking cessation program. It checks in with users twice a day and is available for support when needed. Uses positive reinforcement for smoke-free days, resisting cravings, and quit milestones.	Behavior change techniques
Simon et al [18]	Descriptive summary paper	Chatbots for smoking cessation among adolescents with a low socioeconomic status. Describes interventions in motivational interviewing and CBT, as well as some gaps in the literature and future recommendations.	Motivational interviewing and CBT
Wang et al [25]	Randomized controlled trial	Chatbot on a WeChat support group. The chatbot sends announcements, reminders, ideas, and responses to the support group.	N/A

^a^N/A: not applicable.

^b^CBT: cognitive behavioral therapy.

^c^MAS: multiagent systems.

^d^ACT: acceptance and commitment therapy.

#### Sample Size and Intervention Duration

The pilot studies [20,21] and trials [22,23] had samples that ranged from 6 to 121 participants. With respect to the RCTs, the samples were reasonably large (n=57,214 [24] and n=401 [25]). The intervention duration for the RCTs was relatively short at only 1 to 2 months, and the longest follow-up period was 52 weeks in 1 pilot study [21].

#### Participant Characteristics

Each study included participants who were adult tobacco smokers. These participants were recruited from countries across Europe, America, and Asia. There were no studies in low- and middle-income countries (LMICs).

#### Measures and Use of Theory

Some of the studies used a validated measure, for example, the quality of life scale EuroQol-5D-5L (5-level EuroQol 5-dimensional questionnaire) [19] or the nicotine dependence scale (Fagerstrom Test for Nicotine Dependence) [20,21]. However, a number of studies used self-report measures to assess smoking as the postintervention measure [24,25]. Out of 9 studies, 7 referenced at least one theory. The theories included psychological theories such as motivational interviewing, acceptance and commitment therapy, cognitive behavioral therapy, BCTs, gamification, and problem-solving.

#### Types of Chatbots

The chatbot interventions varied significantly across studies. Some chatbots were described as embodied conversational agents, which used an avatar in the form of a person to talk with the user [17,20,22]. Others used a social media platform, such as WhatsApp [25], or were part of an app on the mobile device [21,24]. The additional studies described the chatbot as a function on its own, which delivered smoking cessation support or delivered a smoking cessation program via a website or mobile device [16,19,23].

### Study Outcomes

#### Smoking Cessation

A number of studies found positive outcomes for smoking cessation measures. Four studies noted improvements in quit rates. Karekla et al [20] found that the treatment group had significantly higher self-reported quit rates compared with controls at 6 months. Masaki et al [21] found that the chatbot intervention led to continuous abstinence rates of 64% from 9 to 24 weeks, 76% from 9 to 12 weeks, and 58% from 9 to 52 weeks. This was as a supplement to an outpatient nicotine dependence service, and these rates were said by the authors to be greater than those in previous studies of the outpatient service. Perski et al [24] also found that those who received a chatbot in support of their existing Smoke Free app had 1.41 times greater odds of being abstinent at 1 month than those who received the standard version of the app without the chatbot. Lastly, Wang et al [25] found that those in the intervention group had a higher quit rate compared with controls at 6 months (20.1% vs 12.4%). Although Almusharraf et al [23] did not measure smoking cessation, 8.3% of participants noted that the intervention helped with their smoking habits. Two studies found that the chatbot intervention led to a reduction in the number of cigarettes smoked per day after 14 days [22] and 6 months [20]. Other reported benefits to smoking cessation included lower levels of nicotine dependence at 6 months [20] and 12 weeks [21].

#### Additional Outcomes

The studies in this scoping review also found some additional benefits worth highlighting. Three studies noted that the intervention was liked by participants. Abdullah et al [22] found that the overall appraisal was very positive, and Karekla et al [20] found that the satisfaction, interest, engagement, acceptability, and helpfulness of the intervention were rated highly. Almusharraf et al [23] found that 34.7% of participants enjoyed the interaction with the chatbot. In addition to these findings, Karekla et al [20] found that their chatbot intervention led to improvements in self-efficacy at 6 months compared with the control group. Masaki et al [21] found improvements in the mood and physical symptom scale at 12 weeks in the intervention group. Lastly, Wang et al [25] found improvements in engagement. When the chatbot was activated, researchers found an increase in the number and types of messages sent by 61%, and those who had not smoked in the past week had a significantly higher number of conversations with the chatbot compared with those who had smoked.

#### Study Quality

A review of research methodology found that most studies were of low quality. Only 2 RCTs were found. In 1 RCT, only 10.7% of the overall sample responded at a 1-month follow-up [24]. The other RCT failed to report any P values [25]. In addition, the follow-up period was short (1 to 2 months). One framework paper highlighted some preliminary findings of a trial; however, the researchers did not provide many methodological details [16], and another trial had an attrition rate of 42% [20].

## Discussion

The aim of this scoping review was to provide a general summary of the extent of evidence for chatbots in smoking cessation. Overall, a summary of the evidence found that there are currently limited high-quality studies assessing chatbots for smoking cessation. Two RCTs provided promising results. However, both had significant methodological and reporting issues [24,25]. The trial and pilot studies also showed efficacy; however, due to the limited power of these studies, generalization to larger populations is limited. This scoping review suggests that larger-scale high-quality studies are needed to assess the effectiveness of chatbots for smoking cessation.

While the publication of reviews on the technical development and effectiveness of chatbots in general continues at pace [26-29], this scoping review highlights a number of key concerns specific to the evidence for the use of chatbots in smoking cessation. First, as discussed, the quality of evidence was low, with issues, such as short-term follow-up, high attrition/low response rates, and poorly described methodology. Another key concern was the absence of studies from LMICs. High-income countries are likely to have a different context, including good tobacco control policies and low smoking prevalence, making the generalizability of the findings of these studies to LMICs problematic. There were also no studies conducted in younger populations who may be more used to or willing to use chatbot technology. Simon et al [18] noted in their paper that smoking cessation interventions are needed for adolescents having a low socioeconomic status. Thus, future research will need to test these interventions in more diverse populations.

The last key concern was regarding the language used to describe chatbot interventions. The way researchers describe interventions varies significantly across studies. For example, some were described as “conversational agents,” “embodied conversational agents,” “digital avatars,” or apps that simply contained a chatbot. This heterogeneity makes it difficult to compare and accurately assess the efficacy of chatbot functionality. With no “agreed” terms and less than full descriptions of the functions these technologies provide, the likelihood of missing interventions that function like chatbots is high.

Further considerations raised by this scoping review include the continuum of chatbot technologies used in interventions. Some are relatively simple, for example, providing a set of responses/questions that participants choose from, which is not very different from existing messaging-based smoking cessation interventions that have been proven effective [30-32], while others are more complex and can respond with highly tailored responses to participants’ individual questions using artificial intelligence methods. Chatbot technologies can be delivered in a variety of ways, from stand-alone apps and being embedded in existing social media/messaging programs to more sophisticated “digital human” technologies (eg, SoulMachines [33]). These variations may potentially impact engagement and effectiveness and thus should be fully described in any reports or studies.

It is also possible that chatbots can be used for other interventions related to, but not directly about, smoking cessation. We found a paper that described chatbots for screening substance users [34]. Chatbots may function well in helping with health-seeking behaviors and accessing health services before providing actual smoking cessation assistance.

While this scoping review provides an effective summary of the current chatbot literature, there are some limitations of this review. We did not conduct a formal assessment of the methodological quality of the literature [14] owing to the obvious lack of sufficient quality trials. There was also considerable heterogeneity across the chatbot interventions with respect to the description, level of contact with the intervention, methodology, and outcome measures. This made it difficult to assess the overall efficacy of chatbots and to synthesize the findings.

Despite these limitations, this review has some significant strengths. This review searched published and grey literature and did not place any restrictions on the types of studies included. As a result, we were able to assess a variety of studies, which provided a good summary of the current state of the research.

It is evident that more high-quality trials need to be performed to fully assess the efficacy of chatbots for smoking cessation. In addition, it is hoped that formal evaluations of smoking cessation chatbots developed by the WHO during the COVID-19 pandemic will provide real-world evidence to contribute to the body of knowledge about the impact of chatbots for smoking cessation.

In conclusion, this scoping review provides a summary of the use of chatbots for smoking cessation. Overall, we found some evidence for effectiveness; however, the number of studies assessing chatbots is limited, and it is evident that more high-quality trials are needed. Future research should aim to provide more in-depth descriptions of chatbot functionality, mode of delivery, and theoretical underpinnings. It should compare chatbots with proven text messaging and other cessation interventions to determine whether they can be more effective than current programs. Finally, authors should use consistent terminology in their descriptions of chatbots and in the keywords to ensure their studies are easily searchable for future reviews.

## Appendix

Multimedia Appendix 1 Details of the included studies.

## Abbreviations

BCT: behavior change technique
LMIC: low- and middle-income country
RCT: randomized controlled trial
WHO: World Health Organization

## References

[1] Tobacco. World Health Organization.

[2] WHO global report on trends in prevalence of tobacco use 2000-2025, fourth edition. World Health Organization.

[3] (2020). WHO statement: Tobacco use and COVID-19. World Health Organization.

[4] Number of smartphone subscriptions worldwide from 2016 to 2021, with forecasts from 2022 to 2027. Statista.

[5] Cuijpers P, van Straten A, Andersson G (2008). Internet-administered cognitive behavior therapy for health problems: a systematic review. J Behav Med.

[6] Domhardt M, Steubl L, Baumeister H (2020). Internet- and mobile-based interventions for mental and somatic conditions in children and adolescents. Z Kinder Jugendpsychiatr Psychother.

[7] Free C, Phillips G, Galli L, Watson L, Felix L, Edwards P, Patel V, Haines A (2013). The effectiveness of mobile-health technology-based health behaviour change or disease management interventions for health care consumers: a systematic review. PLoS Med.

[8] Whittaker R, McRobbie H, Bullen C, Rodgers A, Gu Y, Dobson R (2019). Mobile phone text messaging and app-based interventions for smoking cessation. Cochrane Database Syst Rev.

[9] Michie S, Free C, West R (2012). Characterising the ‘Txt2Stop’ smoking cessation text messaging intervention in terms of behaviour change techniques. J. Smok Cessat.

[10] Michie S, van Stralen MM, West R (2011). The behaviour change wheel: a new method for characterising and designing behaviour change interventions. Implement Sci.

[11] Lucas G, Gratch J, King A, Morency L (2014). It’s only a computer: Virtual humans increase willingness to disclose. Computers in Human Behavior.

[12] Brandtzaeg PB, Følstad A (2017). Why People Use Chatbots. Internet Science. INSCI 2017. Lecture Notes in Computer Science, vol 10673.

[13] Laranjo L, Dunn A, Tong H, Kocaballi A, Chen J, Bashir R, Surian D, Gallego B, Magrabi F, Lau A, Coiera E (2018). Conversational agents in healthcare: a systematic review. J Am Med Inform Assoc.

[14] Tricco A, Lillie E, Zarin W, O'Brien K, Colquhoun H, Levac D, Moher D, Peters M, Horsley T, Weeks L, Hempel S, Akl EA, Chang C, McGowan J, Stewart L, Hartling L, Aldcroft A, Wilson MG, Garritty C, Lewin S, Godfrey CM, Macdonald MT, Langlois EV, Soares-Weiser K, Moriarty J, Clifford T, Tunçalp Ö, Straus SE (2018). PRISMA Extension for Scoping Reviews (PRISMA-ScR): Checklist and Explanation. Ann Intern Med.

[15] Ouzzani M, Hammady H, Fedorowicz Z, Elmagarmid A (2016). Rayyan-a web and mobile app for systematic reviews. Syst Rev.

[16] Calvaresi D, Calbimonte JP, Dubosson F, Najjar A, Schumacher M (2019). Social Network Chatbots for Smoking Cessation: Agent and Multi-Agent Frameworks. WI '19: IEEE/WIC/ACM International Conference on Web Intelligence.

[17] Grolleman J, van Dijk B, Nijholt A, van Emst A, IJsselsteijn WA, de Kort YAW, Midden C, Eggen B, van den Hoven E (2006). Break the Habit! Designing an e-Therapy Intervention Using a Virtual Coach in Aid of Smoking Cessation. Persuasive Technology. PERSUASIVE 2006. Lecture Notes in Computer Science, vol 3962.

[18] Simon P, Krishnan-Sarin S, Huang TH (2019). On Using Chatbots to Promote Smoking Cessation Among Adolescents of Low Socioeconomic Status. arXiv.

[19] Ávila-Tomá J, Espinosa E, Lorenzo C, Suberviola F, Pardo B, Serrano M, Güeto-Rubio M, Grupo Dej@lo (2020). Dejal@Bot: Un chatbot aplicable en el tratamiento de la deshabituación tabáquica. Revista de Investigación y Educación en Ciencias de la Salud (RIECS).

[20] Karekla M, Savvides S, Gloster A (2020). An avatar-led intervention promotes smoking cessation in young adults: A pilot randomized clinical trial. Ann Behav Med.

[21] Masaki K, Tateno H, Kameyama N, Morino E, Watanabe R, Sekine K, Ono T, Satake K, Suzuki S, Nomura A, Betsuyaku T, Fukunaga K (2019). Impact of a novel smartphone app (CureApp Smoking Cessation) on nicotine dependence: Prospective single-arm interventional pilot study. JMIR Mhealth Uhealth.

[22] Abdullah A, Gaehde S, Bickmore T (2018). A tablet based embodied conversational agent to promote smoking cessation among veterans: A feasibility study. J Epidemiol Glob Health.

[23] Almusharraf F, Rose J, Selby P (2020). Engaging unmotivated smokers to move toward quitting: Design of motivational interviewing-based chatbot through iterative interactions. J Med Internet Res.

[24] Perski O, Crane D, Beard E, Brown J (2019). Does the addition of a supportive chatbot promote user engagement with a smoking cessation app? An experimental study. Digital Health.

[25] Wang H, Zhang Q, Ip M, Fai Lau J (2018). Social media–based conversational agents for health management and interventions. Computer.

[26] Rapp A, Curti L, Boldi A (2021). The human side of human-chatbot interaction: A systematic literature review of ten years of research on text-based chatbots. International Journal of Human-Computer Studies.

[27] Wang J, Hwang G, Chang C (2021). Directions of the 100 most cited chatbot-related human behavior research: A review of academic publications. Computers and Education: Artificial Intelligence.

[28] Borsci S, Malizia A, Schmettow M, van der Velde F, Tariverdiyeva G, Balaji D, Chamberlain A (2021). The Chatbot Usability Scale: the design and pilot of a usability scale for interaction with AI-based conversational agents. Pers Ubiquit Comput.

[29] Oh Y, Zhang J, Fang M, Fukuoka Y (2021). A systematic review of artificial intelligence chatbots for promoting physical activity, healthy diet, and weight loss. Int J Behav Nutr Phys Act.

[30] Bramley D, Riddell T, Whittaker R, Corbett T, Lin R, Wills M, Jones M, Rodgers A (2005). Smoking cessation using mobile phone text messaging is as effective in Maori as non-Maori. N Z Med J.

[31] Chen J, Ho E, Jiang Y, Whittaker R, Yang T, Bullen C (2020). Mobile social network-based smoking cessation intervention for Chinese male smokers: Pilot randomized controlled trial. JMIR Mhealth Uhealth.

[32] Rodgers A, Corbett T, Bramley D, Riddell T, Wills M, Lin R, Jones M (2005). Do u smoke after txt? Results of a randomised trial of smoking cessation using mobile phone text messaging. Tob Control.

[33] Overview of Our Digital Brain: How the Soul Machines Digital Brain is autonomously animating Digital People. Soul Machines.

[34] Auriacombe M, Moriceau S, Serre F, Denis C, Micoulaud-Franchi J, de Sevin E, Bonhomme E, Bioulac S, Fatseas M, Philip P (2018). Development and validation of a virtual agent to screen tobacco and alcohol use disorders. Drug Alcohol Depend.

