# Supplementary material for: Chatbots for Smoking Cessation: Scoping Review
Source: J Med Internet Res. 2022 Sep 26;24(9):e35556. doi: 10.2196/35556 (PMC9514452; doi:10.2196/35556)
Supplement: Multimedia Appendix 1 [file jmir_v24i9e35556_app1.docx]

**Multimedia Appendix 1.** Details of the included studies.

| Study | Type of paper | Chatbot intervention | Intervention duration | Participants | Comparison group | Theory | Measures | Results |
| --- | --- | --- | --- | --- | --- | --- | --- | --- |
| Abdullah et al [22] | Study trial | Simple embodied conversational program that encourages smokers to think about setting a quit date. When participants log in, the agent initiates a discussion based on recorded information in the previous session. | 14 days | 6 veteran smokers from the United States | N/A^a^ | N/A | Self-reported questionnaire about smoking and quitting characteristics of participants  Self-reported satisfaction with using the chatbot | Overall appraisal was very positive. Out of 6 participants, 5 reduced the average number of cig­arettes smoked during the last 14 days, and 3 out of 6 had tried to quit. |
| Almusharraf et al [23] | Study trial | Chatbot based on motivational interviewing. The chatbot delivers questions about the pros and cons of smoking. | 1 week | 121 adult tobacco smokers from Canada | N/A | Motivational interviewing | Pros and cons of smoking.  Voluntary feedback | Twenty-one unique reasons for and against smoking were found.  Overall, 34.7% of participants enjoyed the interaction with the chatbot, and 8.3% noted smoking cessation benefits. |
| Avila-Tomas et al [19] | Study protocol | Chatbot that guides users through the stages of the dishabituation process. Includes cognitive-behavioral, relapse-prevention, and problem-solving techniques. | 6 months | 460 adult tobacco smokers in Spain | Standard care | Gamification, cognitive-behavioral theory, problem-solving | Continuous tobacco abstinence (yes/no) at 6 months  Quality of life (EuroQol-5D-5L^b^)  Number of consultations and total time  Cost-utility  Adherence to treatment (number of weeks the patient gets a prescription) | N/A |
| Calvaresi et al [16] | Framework paper | Paper that describes a single-agent and multiagent chatbot based on CBT^c^ and MAS^d^ (underlying technology which models human-like behaviors). | N/A | N/A | N/A | CBT | N/A | Results from the campaign reported at the end: 270 participants (28.9%) succeeded in the smoking cessation goal, 3 months after the last cigarette.  Cessations were 10% higher than the previous edition of the program with no chatbot. |
| Grolleman et al [17] | Framework paper | Describes designing an embodied conversational agent for smoking cessation. | N/A | N/A | N/A | Motivational interviewing and nonverbal listening techniques | N/A | N/A |
| Karekla et al [20] | Pilot randomized clinical trial | Digital avatar–led ACT^e^ smoking cessation program. The program ran for 6 sessions, 25 minutes each. The avatar provides questions, and users answer. Involved ACT-based activities and homework exercises. | 6 months | 84 tobacco smokers who have no interest in quitting from Cyprus | Waitlist control group | ACT | Cessation status  Number of cigarettes smoked per day  Nicotine dependence (FTND^f^)  Self-efficacy (SSEQ^g^)  End of session satisfaction questionnaire | The treatment group had significantly higher self-reported quit rates compared to the control group^h^.  Fewer cigarettes smoked in the treatment condition compared to controls^h^.  The treatment group had lower levels of nicotine dependence compared to controls^i^.  Those in the intervention condition had higher self-efficacy compared to controls^h^.  Satisfaction, interest, engagement, acceptability, and helpfulness of the intervention were positive (rated above 8 out of 10).  Note: attrition was 42%. |
| Masaki et al [21] | Single-arm pilot study | Smoking cessation app called CureApp that includes an artificial intelligence nurse to which users can send a message when they have a craving or are going through withdrawal. The nurse will respond with personalized advice and how to deal with the symptom. | 52 weeks | 56 adult tobacco smokers from Japan | N/A | N/A | Nicotine dependence (FTND)  Mood and physical symptom scale (MPSS)  Tobacco craving questionnaire (FTCQ-12^j^)  Kano test for nicotine dependence (KTSND) | Continuous abstinence rate from 9 to 24 weeks was 64%, 9 to 12 weeks was 76%, and 9 to 52 weeks was 58%.  The mean score decreased by 6.5 points from baseline to 12 weeks.  The mean score decreased by 0.6 points from baseline to 12 weeks.  The mean score decreased by 6.7 points from baseline to week 12.  Note: no *P* values reported. |
| Perski et al [24] | Randomized controlled trial | Smoking cessation chatbot that guides users through the UK “Stop Smoking Services” standard smoking cessation program. It checks in with users twice per day and is available for support when needed. Uses positive reinforcement for smoke-free days, resisting cravings, and quit milestones. | 1 month | 57,214 tobacco smokers from the United Kingdom | The control group received the app without the chatbot function | Behavior change techniques | The total frequency of engagement (number of log ins)  Self-reported continuous abstinence | Compared to the standard version, the Smoke Free app (chatbot included) was associated with a 107% increase in the frequency of engagement^h^ (authors state this is likely to be inflated).  Compared with the standard version of the app, the intervention group had 1.41 times greater odds of being abstinent at 1 month.  Note: only 10.7% of the overall sample responded to the 1-month follow-up. |
| Simon et al [18] | Descriptive summary paper | Chatbots for smoking cessation among adolescents with a low socioeconomic status. Describes interventions in motivational interviewing and CBT, as well as some gaps in the literature and future recommendations. | N/A | N/A | N/A | Motivational interviewing and CBT | N/A | N/A |
| Wang et al [25] | Randomized controlled trial | Chatbot on a WeChat support group. The chatbot sends announcements, reminders, ideas, and responses to the support group. | 2 months | 401 adult tobacco smokers from China | Controls received smoking cessation information and tips | N/A | Numbers and types of messages sent  Self-report smoking questionnaire | An increase of 61% was observed when the conversational agent was activated (no *P* value reported).  Participants who had not smoked in the past week had a significantly higher number of conversations compared to those who smoked^i^.  At another 6-month follow-up, the quit rate in the intervention group (20.1%) was higher compared to controls (12.4%)^i^. |

^a^N/A: not applicable.

^b^EuroQol-5D-5L: 5-level EuroQol 5-dimensional questionnaire.

^c^CBT: cognitive behavioral therapy.

^d^MAS: multi-agent systems.

^e^ACT: acceptance and commitment therapy.

^f^FTND: Fagerström Test for Nicotine Dependence.

^g^SSEQ: Smoking Self-Efficacy Questionnaire.

^h^*P*<.001.

^i^*P*<.05.

^j^FTCQ-12: French versions of the 12-item Tobacco Craving Questionnaire.
